# Systems Pharmacology and *In Silico* Docking Analysis Uncover Association of CA2, PPARG, RXRA, and VDR with the Mechanisms Underlying the Shi Zhen Tea Formula Effect on Eczema

**DOI:** 10.1155/2021/8406127

**Published:** 2021-05-13

**Authors:** Zhen-Zhen Wang, Yuan Jia, Kamal D. Srivastava, Weihua Huang, Raj Tiwari, Anna Nowak-Wegrzyn, Jan Geliebter, Mingsan Miao, Xiu-Min Li

**Affiliations:** ^1^Academy of Chinese Medical Science, Henan University of Chinese Medicine, Zhengzhou 450046, China; ^2^Department of Microbiology & Immunology, New York Medical College, New York 10595, USA; ^3^General Nutraceutical Technology LLC, Elmsford, New York 10523, USA; ^4^Department of Pathology, New York Medical College, New York 10595, USA; ^5^Department of Otolaryngology, School of Medicine, New York Medical College, New York 10595, USA; ^6^Department of Pediatrics, New York University Langone Health, New York, NY 10029, USA; ^7^Department of Pediatrics, Gastroenterology and Nutrition, Collegium Medicum, University of Warmia and Mazury, Olsztyn 10-561, Poland

## Abstract

Eczema is a complex chronic inflammatory skin disease impacted by environmental factors, infections, immune disorders, and deficiencies in skin barrier function. Shi Zhen Tea (SZT), derived from traditional Chinese medicine Xiao-Feng-San, has shown to be an effective integrative therapy for treating skin lesions, itching, and sleeping loss, and it facilitates reduction of topical steroid and antihistamine use in pediatric and adult patients with severe eczema. Yet, its active compounds and therapeutic mechanisms have not been elucidated. In this study, we sought to investigate the active compounds and molecular mechanisms of SZT in treating eczema using systems pharmacology and *in silico* docking analysis. SZT is composed of 4 medicinal herbs, Baizhu (*Atractylodis macrocephalae rhizome*), Jingjie (*Schizonepetae herba*), Kushen (*Sophorae flavescentis radix*), and Niubangzi (*Arctii fructus*). We first identified 51 active compounds from SZT and their 81 potential molecular targets by high-throughput computational analysis, from which we identified 4 major pathways including Th17 cell differentiation, metabolic pathways, pathways in cancer, and the PI3K-Akt signaling pathway. Through network analysis of the compound-target pathway, we identified hub molecular targets within these pathways including carbonic anhydrase II (CA2), peroxisome proliferator activated receptor *γ* (PPAR *γ*), retinoid X receptor *α* (RXRA), and vitamin D receptor (VDR). We further identified top 5 compounds including cynarine, stigmasterin, kushenol, *β*-sitosterol, and (24S)-24-propylcholesta-5-ene-3*β*-ol as putative key active compounds on the basis of their molecular docking scores with identified hub target proteins. Our study provides an insight into the therapeutic mechanism underlying multiscale benefits of SZT for eczema and paves the way for developing new and potentially more effective eczema therapies.

## 1. Introduction

Eczema, characterized by itchy, scaly, erythematous, and oozing skin, is the most common chronic inflammatory skin disorder, affecting 10–20% of children and 1–3% of adults [1, 2]. In addition, allergic diseases such as allergic rhinitis, food allergy, and asthma are frequent comorbidities in moderate-to-severe eczema patients. Eczema severely impacts the life quality of patients and exerts tremendous financial and social burden [3]. In the United States, direct economic costs for the treatment of atopic eczema reached approximately $3.8 billion per year in 2015 [4]. Eczema was ranked first in 2010 global “skin disease burden,” which takes into account severe health loss, including psychological, social, and financial consequences [5]. The pathogenesis of eczema is complicated and is associated with genetic and environmental factors [6]. Severe eczema often fails to respond adequately to therapies specifically targeting a single cytokine or inflammatory mediator. To date, there is no effective cure for eczema, although some treatments can temporarily relieve skin symptoms and reduce inflammation [7]. Thus, the development of new treatments to effectively regulate the immune system and rebuild the skin barrier is an unmet need.

Traditional Chinese Medicine (TCM) has demonstrated significant success in treating complex diseases by regulating multiple pathways working to return dysfunctional organ systems back to normal [8]. Over the past 20 years, multiple studies have reported the efficacy of TCM for the treatment of eczema without serious adverse effects [9, 10]. For example, composite poria granules have shown clinical efficacy in eczema treatment [11]. Recently, Run-Zao-Zhi-Yang capsules (RZZYC) have been reported to provide symptom relief, maintain long-term remission, and improve quality of life, with fewer recurrences [12]. In addition, the well-known famous TCM formula Xiao-Feng-San (XFS) has been used for the treatment of skin disorders since the ancient Ming Dynasty in China [13]. It was reported to be one of the top 5 most commonly prescribed herbal formulas for eczema (total prescriptions = 381,282) in Taiwan [14]. We developed Shi Zhen Tea (SZT), a derivative of XFS that has been successfully used in the US as integrative eczema therapy [8]. It is comprised of four medicinal herbs, *Sophorae flavescentis radix* (Kushen, K), *Schizonepetae herba* (Jingjie, J), *Arctii fructus* (Niubangzi, N), and *Atractylodis macrocephalae rhizoma* (Baizhu, B), which display strong ability of dispersing cold, dispelling wind, and dehumidification. It showed efficacy in treating moderate-to-severe eczema in both children and adults [15]. Disease severity scores decreased in all patients, and most patients stopped taking steroids or antihistamines after the 3-month treatment. However, as with most traditional medicines, its mechanisms of action are elusive, due to presence of multiple, complex compounds, and their metabolites. By combining discovery of new actionable disease targets and isolation of active compounds, the systems pharmacology approach together with high-throughput computational analysis provides a powerful tool to study mechanisms underlying TCM function. A growing body of evidence [16–18] suggests that the use of systems pharmacology can lead to an integrated understanding of drug action on the human interactome which can be used for drug discovery and pharmacological analysis of multiple agents on complex diseases along with prediction of adverse effects. Recently, certain classical Chinese prescriptions, such as Huo-Xiang-Zheng-Qi formula [19], Dan-Shen formula [20], Xiao-Zheng-Fang formula [21], and Bu-Fei-Yi-Shen formula [22], as well as antiasthma herbal medicine intervention (ASHMI), a formula previously developed by us [23], have been analyzed and evaluated by systems pharmacology.

**Figure 1:**
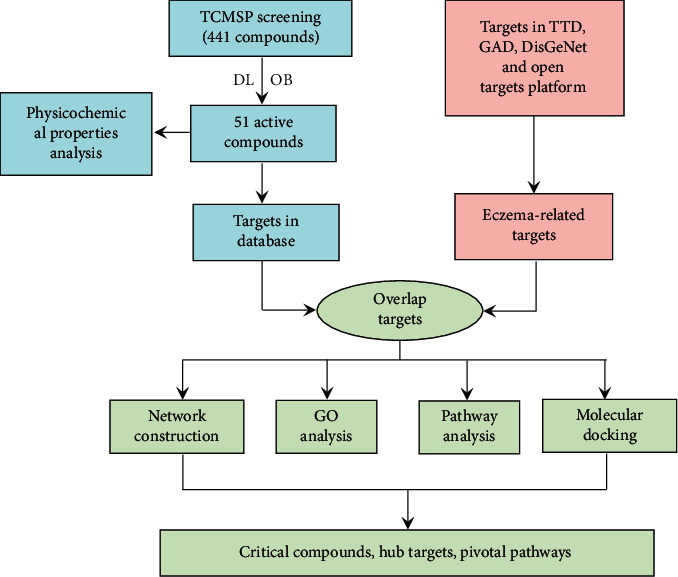
The workflow of systems pharmacology used for analysis of SZT formula for eczema treatment. TCMSP: TCM Systems Pharmacology; OB: oral bioavailability; DL: drug-likeness; TTD: therapeutic target database; GAD: genetic association database; and GO: gene ontology. First, 51 active compounds of formula were selected from the TCMSP database. Their biological targets were further collected from several reliable databases. Meanwhile, physicochemical property analysis was conducted to sum up common properties of active compounds in different physicochemical parameters. Similarly, the targets involved in the pathological process of eczema were collected from related databases including TTD, GAD, DisGeNet, and Open Targets Platform. With biological targets of compounds and related targets of eczema, their shared targets are chosen as potential therapeutic targets of SZT for eczema. Then, network construction, GO analysis, pathway analysis, and molecular docking are used to reveal the critical compounds from the SZT, the hub targets, and pivotal regulated pathway of SZT for eczema treatment.

The current study seeks to uncover the therapeutic mechanisms' underlying efficacy of SZT on eczema using systems pharmacology, through combined evaluation of absorption, distribution, metabolism, and excretion (ADME), compound feature mapping, drug target mining, target enrichment network and pathway analyses, and *in silico* molecular docking. The whole workflow of the study is shown in Figure 1. Results of these analyses will contribute to the understanding of how SZT formula functions, on the molecular level, in the treatment of eczema, providing the rationale and tools to improve the formulation for better TCM treatment efficacy.

## 2. Materials and Methods

### 2.1. Building of the Compound Database

The SZT formula is composed of four Chinese herbs: *Sophorae flavescentis radix* (Kushen, K), *Schizonepetae herba* (Jingjie, J), *Arctii fructus* (Niubangzi, N), and *Atractylodis macrocephalae rhizoma* (Baizhu, B). The compounds of these four herbs were collected from the TCM Systems Pharmacology (TCMSP) database [24].

### 2.2. Active Compound Prediction by Oral Bioavailability and Drug-Likeness Evaluation

Oral bioavailability (OB), one of the vital properties of drugs, is the percentage that is absorbed into the system from an orally administered dose of drugs. The compounds with OB ≥ 30% (20% variability making threshold up to 50%) in TCMSP were selected as active compounds considering the average of OB (30% with 10%–50% variability) in clinical studies [23].

Drug-likeness (DL) is the similarity of a given compound with the physiochemical or/and structural properties of existing drugs, which have been used to evaluate a drug's potential early in the process of discovery. A Tanimoto coefficient was developed to predict the DL value of compounds [25]. The formula is as follows:

(1) $$\begin{array}{c} f\left(A,\;B\right)=\frac{A\cdot B}{{\left|A\right|}^{2}+{\left|B\right|}^{2}-A\cdot B}, \end{array}$$

where *A* represents the molecular properties of herb compounds and *B* represents the average molecular descriptor of molecules from the DrugBank database (drugbank.ca). DL ≥ 0.18 was determined as the criterion to select potential active compounds, where 0.18 was the mean value of DL index in DrugBank [26].

### 2.3. Compound Feature Mapping

The pharmacological activities of a compound are directly related to their physicochemical properties. The physicochemical parameters included in Lipinkski's Rule of Five, including molecular weight, lipohydro partition coefficient, hydrogen donor and acceptor, and rotatable bonds, have been used to evaluate druggability of compounds [27]. Thus, estimation of relevant physicochemical properties of active compounds may provide information about their drug likeness. Thus, the compound feature mapping method [23] was applied to visualize all active compounds from SZT according to the following six physicochemical properties, molecular weight (MW), hydrogen bond donors (nHDon), hydrogen bond acceptor (nHAcc), octanol/water partition coefficient (AlogP), topological polar surface area (TPSA), and number of rotatable bonds (RBN). All six properties were analyzed by principal component analysis (PCA), where six-dimensional data were reduced to two-dimensional data with acceptable former descriptor variation in order to best display distribution of active ingredients in chemical space.

### 2.4. Target Fishing and Classification

First, a simplified molecular-input line-entry system (SMILES) of potential active compounds was prepared by ALOGPS 2.1 program (vcclab.org/web/alogps/). Target predictions were conducted using the SMILES of potential compounds through several published databases, including TCMSP [24], HitPick (mips.helmholtz-muenchen.de/hitpick/), Swiss Target Prediction [28] (swisstargetprediction.ch/), Similarity Ensemble Approach (SEA, sea.bkslab.org/) [29], PubChem (pubchem.ncbi.nlm.nih.gov/) [30], and DrugBank [31]. All the predicted targets obtained from these databases were mined in prevailing databases as follows to select targets for eczema: therapeutic target database (TTD, db.idrblab.net/ttd/) [32], genetic association database (GAD, geneticassociationdb.nih.gov/), DisGeNet (disgenet.org/) [33], and Open Targets Platform (targetvalidation.org/) [34]. Selected targets were finally mapped to the UniProt Database (uniprot.org/) [35] for normalization.

### 2.5. Gene Ontology and Pathway Analysis

Target enrichment gene ontology (GO) and pathway analyses lead us to gain mechanistic insight from the molecular level into biological function level. GO was introduced by mapping targets to the DAVID database (david.ncifcrf.gov/) [36]. The GO biological process terms with false discovery rate (FDR) < 0.01 were selected. Pathways were obtained by mapping targets to KOBAS 3.0 (kobas.cbi.pku.edu.cn) [37]. The significant pathways with FDR <0.01 were selected.

### 2.6. Network Construction and Analysis

With predicted targets of compounds and significant pathways, we built two biological networks using Cytoscape (v3.2.1). The compound-target-disease (C-T-D) network, containing active compounds, their related targets, and disease, provides general information about the pharmacological mechanism of SZT formula in the molecular level. The principal pathways, extracted with its related targets and active compounds, link the pathways to candidate compounds through targets and further interweave a compound-target-pathway-disease (C-T-P-D) network. The properties of these two networks were validated by NetworkAnalyzer [38], a plugin of Cytoscape.

### 2.7. Molecular Docking

To explore the binding modes and offer more insights into the interaction between molecular targets and compounds, molecular docking was performed on hub targets and all related bioactive compounds by AutoDock Vina [39]. Protein crystal structures with excellent resolution were downloaded from RCSB protein data bank (rcsb.org/) [40]. The structures of the ligand were directly downloaded from PubChem (pubchem.ncbi.nlm.nih.gov/) [30] without further optimization. Proteins and compounds were prepared by AutoDockTools (v1.5.6) [41]. The three-dimensional molecular graphics were prepared by the PyMOL system [42] (pymol.org) and Discovery Studio [43]. Generally, all hydrogens and Gasteriger charges were added to each molecule. Docking areas and Autogrid parameters were set based on the binding pockets of proteins. The lowest binding energy was selected and illustrated as the best binding conformation.

## 3. Results and Discussion

### 3.1. Active Compounds Obtained from SZT

In total, 441 compounds from the four herbs of SZT were obtained from TCM Systems Pharmacology (TCMSP), of which 159 were from Jingjie, 113 from Kushen, 55 from Baizhu, and 114 from Niubangzi. Such complexity of SZT makes it difficult to explore its dominant compounds and uncover the biological mechanism of formula. Thus, it is necessary to determine potentially active ingredients' database from the SZT formula. Many compounds found in TCM, while being biologically active, may lack the absorption and bioavailability necessary to overcome barriers during the oral administration process and reach the targets, making it difficult to investigate the mechanisms of herbs. Poor oral bioavailability (OB) may result in low efficacy and cause a new drug to fail in clinical trials. High OB is a critical parameter to evaluate the possibility of a molecule becoming a drug [44]. We used oral bioavailability (OB) and drug-likeness (DL) as criteria to select the compounds as follows. OB ≥ 30% and DL ≥ 0.18 were used as threshold to narrow down the compound scope, based on the theory that drugs require proper physicochemical properties to cross various barriers and play their pharmacological effects. This threshold was determined on account of not only extracting sufficient information from formula but also keeping the balance of active compound numbers from different herbs.

As a result, 51 active compounds from 441 were selected for further analysis. Abbreviation, chemical name, molecular structure, property, and species of each active compound are listed in Table 1, which included flavonoid (25.5%), alkaloid (25.5%), phenylpropanoid (15.7%), terpene (13.7%), steroid (11.7%), and others (7.9%). Selected compounds with satisfactory OB and DL value were assumed as potential active compounds. Among them, 25 compounds (OB ≥ 50% and DL ≥ 0.18) are from 113 compounds of herb Kushen. For example, matrine (K15, OB = 63.77% and DL = 0.25), one of the major tetracycloquinolizindine alkaloids, was reported to exhibit anti-inflammatory [45], antiviral [46], and antiallergic properties [47]. Flavone norkurarinol (K2, OB = 51.28% and DL = 0.64) showed various biological activities such as antioxidant, antibacterial, anti-influenza, and anti-inflammatory activities [48]. For herb Jingjie, 11 of 159 compounds met the filter criteria (OB ≥ 30% and DL ≥ 0.18). Luteolin (J1, OB = 36.16% and DL = 0.25), a flavone present in many herbs, has been proven to inhibit inflammation responses [49]. Quercetin (J2, OB = 46.43% and DL = 0.28), a well-known free-radical scavenger, can stimulate the immune system by decreasing the production of proinflammatory cytokines, suppressing IL-4 production, improving Th1/Th2 balance, and restraining the formation of antigen-specific IgE antibody [50]. In addition, phytosterols, such as *β*-sitosterol (J3, OB = 36.91% and DL = 0.75), sitosterol (J4, OB = 36.91% and DL = 0.75), and stigmasterol (J5, OB = 43.83% and DL = 0.76) have been the subject of increased interest for potential treatment of atopic dermatitis (AD) to replace steroid therapy [51]. For herb Niubangzi, 8 active compounds from 114 were identified to show OB ≥ 30% and DL ≥ 0.18. Especially, arctiin (N2, OB = 34.45% and DL = 0.84), one of the major active ingredients, can be biotransformed to its more potent aglycone arctigenin by intestinal microbiota [52]. Cynarin (N8, OB = 31.75% and DL = 0.68), an ester of quinic acid and two molecules of caffeic acid, have been proved to display good antioxidant, antiradical, and anticholinergic effects [53]. Moreover, *β*-sitosterol (J3, N3, OB = 36.91% and DL = 0.75) and supraene (J6, N5, OB = 33.55% and DL = 0.42) are shared active compounds by both Niubangzi and Jingjie. For herb Baizhu, 7 active compounds from 55 ingredients were selected to meet the criterion (OB ≥ 30% and DL ≥ 0.18). Among them, *α*-amyrin (B3, OB = 39.51% and DL = 0.76), a pentacyclic triterpene, shows potential anti-inflammatory effects [54]. In particular, it significantly suppresses the scratching behavior in a mouse model of pruritus by inhibiting degranulation of mast cell [55], beneficial for skin lesions in eczema.

### 3.2. Compound Feature Mapping Based on Physicochemical Parameters

**Figure 2:**
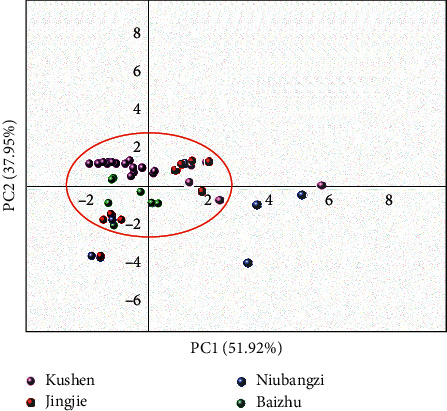
Chemical space distribution of active components present in Kushen, Jingjie, Niubangzi, and Baizhu, based on principal component analysis of their drug-related physicochemical properties. Active compounds in the red circle display a similar physiochemical property.

The drug-like physicochemical properties of active compounds were investigated by analyzing six common drug-associated physicochemical parameters including molecular weight (MW), hydrogen bond donors (nHDon), hydrogen bond acceptor (nHAcc), octanol/water partition coefficient (AlogP), topological polar surface area (TPSA), and number of rotatable bonds (RBN). Principal component analysis (PCA) was applied to display the distribution of active compounds in chemical space considering all six physicochemical parameters. The first principle component (PC1) displays the direction of maximal variance which takes 51.92% of total variance, and PC2 represents the second largest variations that account for 37.95% (Figure 2). These two principle components account for 89.87% of the total variances, indicating that they sufficiently represent the six physicochemical features of these compounds. Figure 2 illustrates the relatedness distribution of the active compounds. Most of the active compounds from SZT are clustered in the red circled area, indicating they have similar physicochemical features. It is noted that the binding of compounds to their targets are up to the structure and property of compounds. The result implies that the targets of these active compounds, derived from different herbs but with similar physicochemical properties, may be associated and overlapping.

### 3.3. Potential Target Fishing

Establishing compound-target interactions and enriching those in signaling pathways become increasingly necessary for explicating the mechanism of drug action and their underlying pharmacological effects. The targets of 51 active compounds were fished by several popular databases, including HitPick, Swiss Target Prediction, Similarity Ensemble Approach (SEA), PubChem, and DrugBank. The integrated targets were further mined in targets selected from the therapeutic target database (TTD), genetic association database (GAD), DisGeNet, and Open Targets Platform with terms “eczema” and “atopic dermatitis.” Finally, a total of 81 potential targets were screened out for 51 active compounds with 327 interactions. Most compounds were found to interact with more than one target for eczema, which implies comprehensive regulation and extensive pharmacological actions of SZT. For example, phaseolin (K24) from Kushen interacts with 15 potential targets related to eczema, while kaempferol (N4) from Niubangzi links to 12 potential targets. Among these targets, vascular endothelial growth factor A (VEGFA) [56], epidermal growth factor receptor (EGFR) [57], and interleukin 2 (IL-2) [58] are believed to play crucial roles during the pathogenesis of AD. *β*-sitosterol (N3, J3) from both Niubangzi and Jingjie targets 11 proteins, among which peroxisome-proliferator-activated receptor (PPAR) family and retinoic-acid-receptor-related orphan receptors (RORs) might be the main regulated targets of *β*-sitosterol [59]. RORs regulate the function of specific subsets of T cells and innate lymphoid cells, which are key drivers of inflammatory disease in barrier tissues [60]. Moreover, the retinoid X receptor (RXR) is an intriguing and essential member of nuclear receptors activated by 9-cis retinoic acid to regulate cell differentiation, metabolism, and cell apoptosis [61]. The strong reduction of retinoid signaling and retinoid concentration in affected as well as nonaffected skin of individuals with AD has been discovered, compared to healthy individuals [62]. A selective activator of RAR or RXR may influence the retinoid signaling in skin, and RORA and RORC have been reported to be a potential critical therapeutic target for treating skin diseases of inflammatory etiology [63].

C-T-D network construction is used to display the active compounds and potential targets of SZT for eczema treatment.

#### 3.3.1. Network Analysis

**Figure 3:**
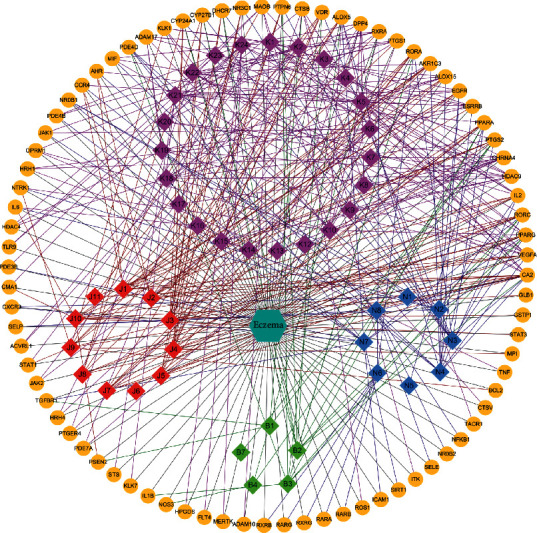
Compound-Target-Disease (C-T-D) network of the SZT formula for eczema treatment. Diamonds, circles, and turquoise hexagon represent bioactive compounds from SZT, corresponding targets associated with eczema, and focused disease eczema. Purple, red, blue, and green diamonds are active compounds from Kushen, Jingjie, Niubangzi, and Baizhu, respectively. Purple, red, blue, and green lines stand for target interactions between Kushen, Jingjie, Niubangzi, and Baizhu, respectively. Black lines indicate relationships between targets and disease eczema. The average degree of networks is 6.3.

Active compound and target data extracted above were employed to construct a C-T-D network containing 129 nodes and 406 edges, involving 47 active compounds and 81 potential targets. Four active compounds K11, K25, B5, and B6 shared no targets with eczema and were, thus, excluded. The network demonstrates multi-interactions between compounds and targets (Figure 3). The diamonds and circles represent active compounds from SZT and their potential targets, respectively. Of 81 targets, Kushen, Jingjie, Niubangzi, and Baizhu recognized 52, 35, 43, and 19 targets, respectively. To evaluate the importance of each node in this network, the degree of each node was defined as the number of edges connected with this node, representing the influence of the node. The network average degree of node was 6.3.

#### 3.3.2. Target Analysis

Most of the targets from our network construction are closely associated with the pathogenesis of eczema. We believe that targets with highest degree are the most vital ones regulated by SZT, thus chosen as hub targets for further analysis. Interestingly, carbonic anhydrase II (CA2) had the largest degree, hit by 17 compounds (5 of Kushen, 3 of Niubangzi, 7 of Jingjie, and 1 of Baizhu). CA2 monitors pH regulation, water transport, and hydration of CO_2_, which is related to diverse diseases such as glaucoma, tumor, epilepsy, and diabetes [64]. CA2 inhibitors, such as acetazolamide and brinzolamide, have been developed and established to treat glaucoma, seizure disorder, and acute mountain sickness [65]. It was reported that CA2 was upregulated by Th2 cytokines in lesion skin of AD patients, which caused a pH rise in skin [66]. The higher surface pH may affect the integrity and cohesion of skin and compromise barrier function of skin in AD [67]. Thus, the CA2 inhibitor may relieve a series of symptoms in dermatitis triggered by itching [68]. Moreover, VEGFA was targeted by 12 active compounds (6 of Kushen, 4 of Jingjie, and 2 of Niubangzi). VEGFA displays a key role in vascular permeability, vasodilation, and angiogenesis. VEGFA can be secreted by various immunocytes in inflamed skin, such as mast cells, macrophages, eosinophils, basophils, and Th17 cells, which appear critical not only in lymphatic vessel expansion but also in antigen clearance and inflammation resolution through enhancement of lymphangiogenesis [56]. Meanwhile, 12 compounds from SZT were found to interact with PPARG. PPARG belongs to a subfamily of nuclear hormone receptors and plays a distinct physiological role in regulating expression of genes involved in cellular proliferation, specific components of the Th2 inflammatory pathway, and maintenance of the skin barrier [69].

Gene ontology (GO) and pathway analysis reveal potential regulation of SZT in the immune, inflammatory, and metabolic processes.

**Figure 4:**
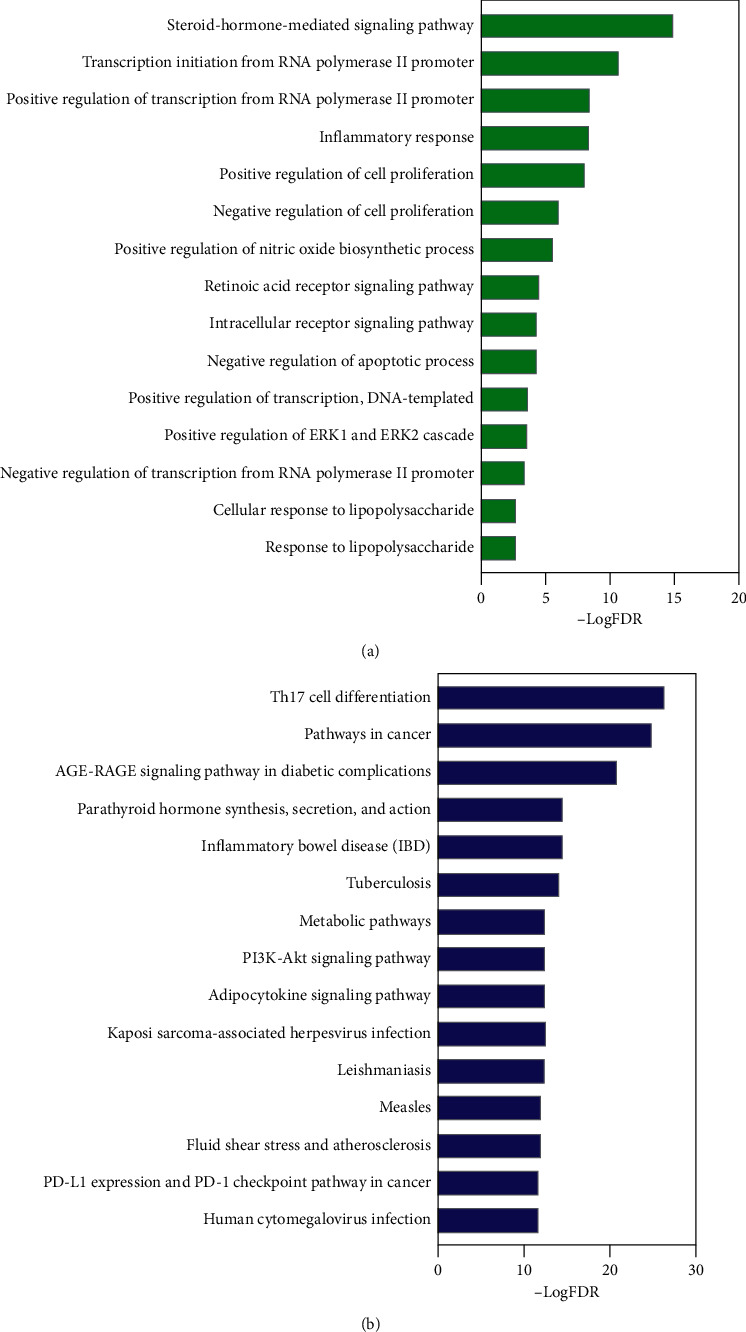
Gene ontology (GO) and pathway analyses. (a) GO biological process analysis of the targets. *Y*-axis: top 15 biological processes relevant to the enriched targets; *X*-axis: significance of each term ranked with −log(false discovery rate) (FDR). (b) Pathway analysis of the targets. *Y*-axis: top 15 significant canonical pathways relevant to the enriched targets; *X*-axis: significance of each pathway ranked by −log(FDR).

Utilizing the identified targets as an enriched gene set, we employed gene-set enrichment analysis to find GO biological process terms in the DAVID database and pathways in the KOBAS database. The top 15 biological process GO terms with False Discovery Rate (FDR) < 0.01 are ranked by enrichment score (–logFDR) in Figure 4(a). FDR is to conceptualize the rate of type I errors in null hypothesis testing when conducting multiple comparison [70]. Most targets are found to be closely related to several biological processes, such as the steroid-hormone-mediated signaling pathway, inflammatory response, retinoic acid receptor signaling pathway, intracellular receptor signaling pathway, and cellular response to lipopolysaccharide. Moreover, most biological process terms listed have been shown to be highly associated with the immunity, inflammation, and/or eczema. In affected or nonaffected skin of patients with AD, the retinoid transport, synthesis, concentration, and signaling were reported to be strongly decreased, suggesting the intrinsic influence of the retinoid signaling pathway to the pathological process of AD [71]. Particularly, the steroid-hormone-mediated signaling pathway with hub targets, such as PPARG, RXRA, RORC, and VDR, is an important regulatory pathway in immunity and inflammation [72]. Inflammatory response, positive regulation of the nitric oxide biosynthetic process, and response to lipopolysaccharide are strongly associated with the inflammatory reaction of the body. The GO results imply that SZT might treat eczema by integral regulating the immune and inflammatory biological processes.

A total of 137 pathways with FDR <0.01 were obtained in KOBAS. The top 15 most significant pathways are listed in Figure 4(b). Most pathways are strongly associated with the immune process, metabolic process, and complex pathways in combination of both. For instance, Th17 cell differentiation, inflammatory bowel disease, tuberculosis, kaposi-sarcoma-associated herpesvirus infection, and human cytomegalovirus infection are more inclined to the immune response induced by internal and/or external stimuli, while the AGE-RAGE signaling pathway in diabetic complications, metabolic pathways, and adipocytokine signaling pathway are more prone to the mediation of the metabolic process in the body. Moreover, both pathways in cancer and the PI3K-Akt signaling pathway are complex and are related to both immune and metabolic processes. Compared with biological process GO analysis, KEGG pathway enrichment provided more detailed information about the signal transmission during biological processes. Thus, key pathways were selected to establish further the C-T-P-D network to determine the relatively critical compounds and hub targets.

A C-T-P-D network was established to reveal the relationships between compounds, targets, pathways and disease.

**Figure 5:**
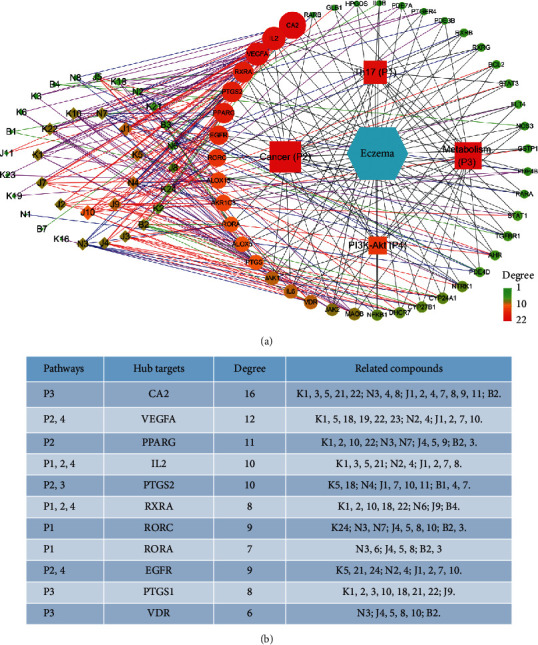
Compound-Target-Pathway-Disease (C-T-P-D) network of the SZT formula for eczema treatment. (a) C-T-P-D network. Square block: the important pathway regulated by SZT; diamonds and circles: bioactive compounds and corresponding targets associated with the four pathways. Turquoise hexagon: disease eczema. Purple, red, blue, and green lines: target interactions between Kushen, Jingjie, Niubangzi, and Baizhu, respectively. Black line: the interaction between targets and the related pathways; the relationship between targets and disease eczema. Node color from green to red and node size are proportional to its degree. (b) Relationship table for pathways, hub targets, and compounds.

We speculate that hub targets with high degree in the network are the most promising targets regulated by SZT. Thus, based on the number of hub targets contained in these pathways, four key pathways were selected, including the Th17 cell differentiation pathway (P1), pathways in cancer (P2), metabolic pathways (P3), and PI3K-Akt signaling pathway (P4). Then, the compounds and targets involved with these four pathways were chosen to construct the C-T-P network, as shown and listed in Figure 5(a). Diamonds, circles, triangles, and hexagons represent bioactive compounds, corresponding targets, pathways, and disease eczema, respectively. Node color from green to red and node size are proportional to its degree in the C-T-P-D network. The network average degree of node was 7.6, with the degree defined as the number of connections. Moreover, detailed information including the involved hub targets, related pathways, and potential ligands is displayed in Figure 5(b). The imbalance of Th1 and Th2 in the pathogenesis of eczema has been widely investigated [73]. Recently, the crucial role of Th17 cell in allergic contact dermatitis has also been demonstrated by several studies. Increasing Th17 activation was confirmed in Asian and pediatric AD [74, 75]. Once activated, Th17 cells have the capacity to produce IL-17A, IL-17F, IL-22, and IL-26. In particular, IL-17 leads to the expression of proinflammatory cytokines, responsible for eosinophil- and neutrophil-mediated inflammation [76]. We found that 16 targets were involved in the Th17 cell differentiation pathway, including hub targets IL-2, RORC, RORA, and RXRA. Among them, IL-2 is released by activated Th1 cells to regulate inflammation and tissue damage. The regulatory roles of RORs and RXRs in the function of lymphoid cell have already been discussed previously [60, 61]. Pathways in cancer, including the MAPK signaling pathway, Jak-STAT signaling pathway, calcium signaling pathway, estrogen signaling pathway, and VEGF signaling pathway, were all identified in the pathway analysis. The hub targets, such as Prostaglandin-Endoperoxide Synthase 2 (PTGS2), EGFR, RXRA, PPARG, VEGFA, and IL-2, were all included in this complex pathway, pathway in cancer. PTGS2, also known as cyclooxygenase-2 (COX-2), is currently the widely used anti-inflammation target of nonsteroidal anti-inflammatory drugs. The inhibition of PTGS2 results in preferable anti-inflammatory effects [77]. In addition to immune function, the association between AD and metabolic syndrome has attracted more attention [78]. Metabolic syndrome includes abdominal obesity, hypertension, insulin resistance, and dyslipidemia [79]. Collective data indicate that central obesity is positively associated with AD, which is likely linked to low-grade systemic inflammation induced by adipocytes (leptin and adiponectin) generated by visceral adipose tissue [80]. CA2, VDR, PTGST1, and PTGST2 are all included in metabolic pathways. The PI3K-Akt signaling pathway is involved in a wide variety of cellular processes, including cellular growth, migration, and proliferation, especially the process of inflammation and immune response. It was reported that the expression of PI3K and Akt in blood was significantly higher in AD patients than in the healthy controls, which might be associated with the intrinsic activation of T-cell and cytokine secretion [81]. Moreover, specific inhibition of PI3K expression led to significant inhibition of T-cell proliferation and secretion of cytokines, such as IL-6 and IL-10. The PI3K-Akt signaling pathway has been reported to be associated with LPS-induced acute inflammatory responses and autophagy regulation in immune response [19].

### 3.4. Molecular Docking Analysis Investigates the Binding Activities

**Figure 6:**
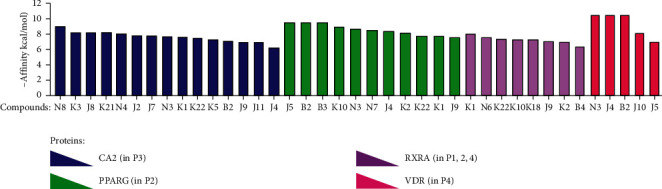
Molecular docking results between proteins and promising ligands. Blue bars: docking affinity between CA2 and 15 potential ligands; green: PPARG and 11 potential ligands; purple: RXRA and 8 potential ligands; and red: VDR and 5 potential ligands.

**Figure 7:**
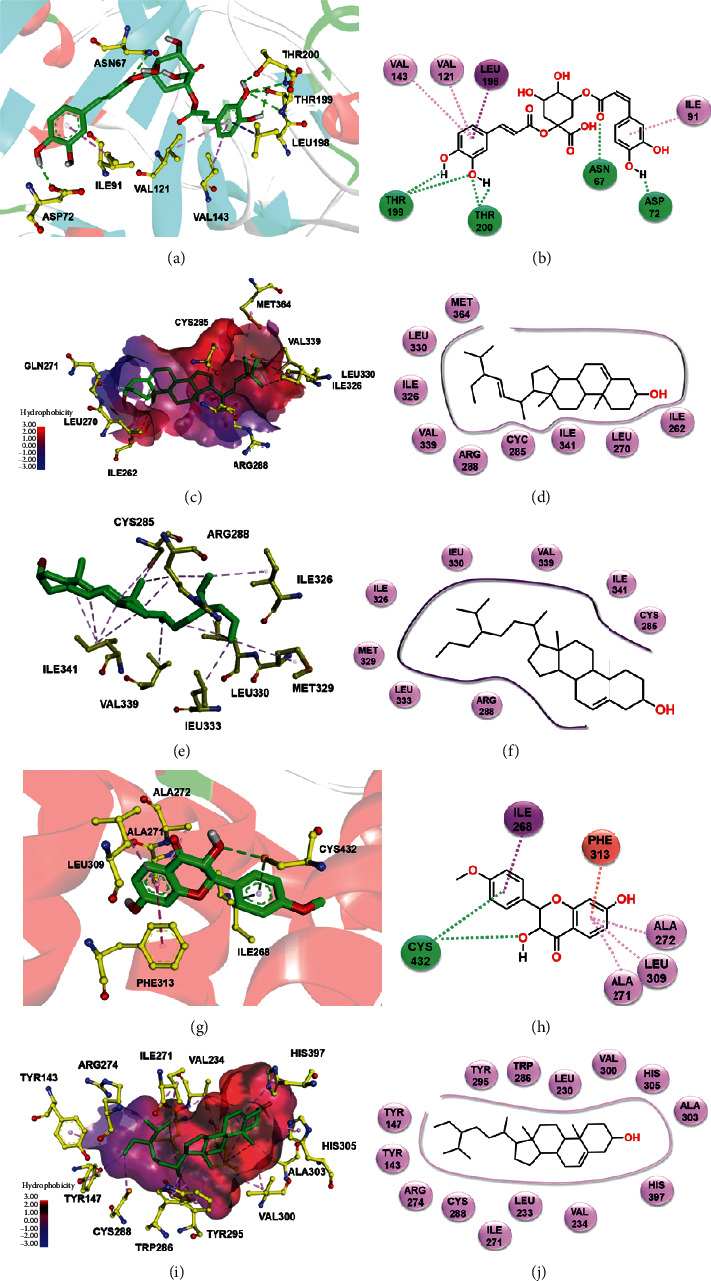
Binding explorations of complex CA2-N8, PPARG-J5, PPARG-B2, RXRA-K1, and VDR-N3. (a) Predicted binding mode of CA2 (PDB: 2Q38) with lowest-energy docking ligand N8 in the active pocket. (b) Two-dimensional (2D) binding view of N8 with CA2. (c) Hydrophobic discrepancy of compound J5 with PPARG (PDB: 2PRG) in the active pocket. (d) 2D binding view of J5 with PPARG. (e) Hydrophobic discrepancy of compound B2 with PPARG in the active pocket. (f) 2D binding view of B2 with PPARG. (g) Predicted binding mode of RXRA with K1 in the active pocket (PDB: 1MVC). (h) 2D binding view of K1 with RXRA. (i) Hydrophobic discrepancy of compound N3 with VDR (PDB: 3A3Z) in the active pocket. (j) 2D binding view of N3 with VDR. Left panel: the carbon and oxygen of ligands are highlighted in green and red, respectively. The yellow, red, and blue binding residues stand for C, O, and N, respectively. Right panel: the green, pink, and purple stand for hydrogen binding, *π*-*π* stacking, andhydrophobic interaction between ligand and related residents, respectively.

To investigate binding between potential targets and active compounds, *in silico* molecular docking was used to calculate the binding energy and evaluate the binding mode between the compound and its target. From hub targets, CA2 (P3), PPARG (P2), and RXRA (P1, P2, and P4) were selected to represent the four different pathways. Additionally, activation of VDR causes marked induction of skin barrier genes and antimicrobial peptide genes in lesion skin, ameliorating allergen-triggered eczema in the murine AD model [82]. Also, it is a potentially novel therapeutic target for treatment of eczema. Thus, molecular docking was conducted for CA2, PPARG, RXRA, and VDR and their interacting active compounds using AutoDock Vina, considering proteins as rigid molecules. For CA2, promising ligands are docking into receptor-binding-domain as inhibitors. Also, for PPARG, RXRA, and VDR, promising ligands are calculated as their agonists. The ligands and their molecular dockings were demonstrated in Figure 6. The results revealed that all compounds as predicted ligands show moderate to strong binding affinity with related proteins. In particular, compounds cynarine (N8), stigmasterol (J5), (24S)-24-propylcholesta-5-ene-3*β*-ol (B2), kushenol (K1), and *β*-sitosterol (N3) had the strongest binding affinities to their receptors. The binding modes of complexes CA2-N8 (−9.1 kcal/mol), PPARG-J5 (−9.3 kcal/mol), PPARG-B2 (−9.2 kcal/mol), RXRA-K1 (−8.1 kcal/mol), and VDR-N3 (−10.6 kcal/mol) are shown in Figure 7.

For CA2, 15 ligands were docked into the CA2 active site and the cynarine (N8) showed highest binding affinity. The hydrophobic pocket formed by Val121, Val143, and Leu198 of CA2 were also observed in the binding of CA2-N8. The hydrogen bond formed between the ligand and Thr199, Thr200, Asn67, and Asp72 further increases the stability of the ligand in the binding site (Figures 7(a) and 7(b)). For PPARG, both stigmasterol (J5) and (24S)-24-propylcholesta-5-ene-3*β*-ol (B2) were well fitted into the binding cavity of the protein by hydrophobic interaction. The binding pocket of PPARG-stigmasterol (J5) shown in Figures 7(c) and 7(d) indicates that nine amino acid residues from PPARG interacted with J5 by hydrophobic interactions, including Leu330, Ile326, Val339, Arg288, Cyc285, Ile341, Leu270, and Ile262. Both stigmasterol and (24S)-24-propylcholesta-5-ene-3*β*-ol are phytosterols with similar chemical structure. The binding energy of PPARG-B2 (Figures 7(e) and 7(f)) is −9.2 kcal/mol, very close to that of PPARG-stigmasterol (J5) (−9.3 kcal/mol). Moreover, the binding of PPARG-N3 (*β*-sitosterol) has previously been studied [83] with similar affinity. For RXRA, docking ligands into the ligand-binding domain was used to evaluate the possibility of these compounds acting as agonists. The strongest binding existed between RXRA and kushenol (K1). Hydrogen binding (Cys432), *π*-*π* stacking (Phe313), and hydrophobic interactions (Ile268, Ala271, Ala272, and Leu309) formed between protein residues and ligand kushenol (K1) were illustrated in Figures 7(g) and 7(h). VDR is a member of nuclear receptor superfamily of transcriptional regulators, which can be activated by its specific ligand vitamin D_3_. The docking result indicated that binding conformation of *β*-sitosterol (N3) fitted well in binding domain of VDR crystallographical structure forming stable complex VDR-N3 (*β*-sitosterol). All 14 amino acid residues showed hydrophobic interactions with ligand N3 (*β*-sitosterol) (Figures 7(i) and 7(j)). Their forceful interaction might attribute to the similar structure of vitamin D_3_ and *β*-sitosterol. Inspiringly, it has been reported that *β*-sitosterol promoted the immune function of vitamin D_3_ [84], suggesting the anti-inflammation and immune enhancement function of *β*-sitosterol. The abovementioned strong interactions further imply the functioning route of SZT compounds through proteins and their corresponding biological activities.

## 4. Conclusions

Eczema is a chronic inflammatory condition causing pruritus, erythema, dryness, scaling, and vesiculopapular rash that negatively impact the quality of life of the affected patients [1]. With no definite cure for eczema, common medicines, such as oral antihistamines, topical corticosteroids, emollients, and even immune-modulating agents, can not only relieve symptoms but also sometimes cause severe side effects [85]. Significantly, these agents do not target upstream molecular dysregulation that is central to eczema. There is an urgent need to develop new treatments to not only relieve symptoms but also address the underlying molecular mechanisms that drive the disease to potentially cure eczema with fewer side effects. TCM has a long and well-known history in Asia treating diseases in a multicompound-multitarget modality [86]. TCM as a complementary or alternative medicine is gradually becoming more widely accepted throughout the world, and molecular basis of its activity is only recently being elucidated [87]. XFS is an acclaimed TCM formula in China documented in “Orthodox Manual of External Medicine” [13], which eliminates itching in the body. We modified the formula to retain four pivotal herbs to develop SZT that has excellent efficacy in treatment of eczema. However, molecular mechanisms of SZT remain poorly defined. Recently, systems pharmacology based on network analysis has provided a new method to uncover the mechanisms of complex formula in TCM [16–18]. We explored the molecular mechanisms of SZT for eczema treatment using a systems pharmacology approach, including ADME evaluation, herb feature mapping, drug target mining, network and pathway analyses, and *in silico* molecular docking.

In summary, we found 51 active compounds out of 441 compounds that were retrieved based on OB and DL screening with satisfactory pharmacokinetic properties. We further studied these active compounds for their physicochemical properties and drug-likeness. Findings of drug target mining supported that active compounds targeted single or multiple proteins involved in the pathological process or treatment of eczema. The C-T-D network analysis, GO, and pathway analyses conducted using DAVAD and KOBAS databases allowed us to elucidate the pharmacological effects of SZT from a holistic perspective. Our results imply that SZT efficacy in eczema may be attributed to immune and metabolic functions via regulation of multiple pathways and networks. Among these, four key pathways stood out, including Th17 cell differentiation, pathways in cancer, metabolic pathways, and PI3K-Akt signaling pathway. Moreover, the C-T-P-D network highlighted the critical active compounds. Finally, molecular docking studies between active compounds and proteins CA2, PPARG, RXRA, and VDR confirmed moderate to strong binding affinities. These data further support the potential biological activities of these active compounds. Overall, the formula affects a regulation of the immune system and metabolic processes, instead of a single target or a single pathway. In vitro and in vivo studies are currently in progress to investigate, confirm, and expand knowledge into the molecular alterations induced by SZT active compounds. In conclusion, systems pharmacology was applied in this work to shed light on the molecular mechanism and pharmacological regulation of SZT for eczema treatment. Our results will help further optimize SZT and facilitate development of more effective drugs to cure eczema.

## Figures and Tables

**Table 1:** Basic information of active compounds from SZT. F, A, P, T, S, and O stand for flavonoid, alkaloid, phenylpropanoid, terpene, steroid, and others, respectively.

Herb	Comp. ID	Comp. name	Mol. Wt.	O.B.	D.L.	Structure	Sp.
Kushen	K1	kushenolJ_qt	286.3	50.86	0.24	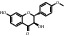	F
Kushen	K2	Norkurarinol	442.55	51.28	0.64	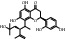	F
Kushen	K3	KushenolJ	580.59	51.39	0.74	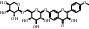	F
Kushen	K4	(+)-Lupanine	248.41	52.71	0.24	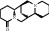	A
Kushen	K5	Norartocarpetin	286.25	54.93	0.24	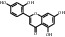	F
Kushen	K6	Sophranol	264.41	55.42	0.28	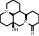	A
Kushen	K7	(+)-Lehmannine	246.39	58.34	0.25	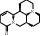	A
Kushen	K8	(+)-Allomatrine	248.41	58.87	0.25	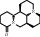	A
Kushen	K9	Sophoridine	248.41	60.07	0.25	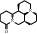	A
Kushen	K10	Leachianone,g	356.4	60.97	0.4	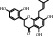	F
Kushen	K11	Isosophocarpine	246.39	61.57	0.25	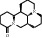	A
Kushen	K12	Anagyrine	244.37	62.01	0.24	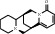	A
Kushen	K13	Leontalbinine	246.39	62.08	0.25	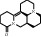	A
Kushen	K14	Lehmanine	246.39	62.23	0.25	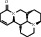	A
Kushen	K15	Matrine	248.41	63.77	0.25	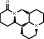	A
Kushen	K16	Sophocarpine	246.39	64.26	0.25	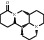	A
Kushen	K17	13,14-Dehydrosophoridine	246.39	65.34	0.25	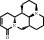	A
Kushen	K18	Inermin	284.28	65.83	0.54	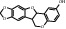	P
Kushen	K19	cis-Dihydroquercetin	304.27	66.44	0.27	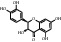	F
Kushen	K20	Isomatrine	248.41	68.68	0.25	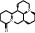	A
Kushen	K21	Formononetin	268.28	69.67	0.21	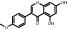	F
Kushen	K22	K22	256.27	71.12	0.18	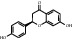	F
Kushen	K23	Inermine	284.28	75.18	0.54	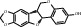	P
Kushen	K24	Phaseolin	322.38	78.2	0.73	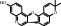	P
Kushen	K25	Glyceollin	338.38	97.27	0.76	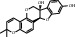	P
Jingjie	J1	Luteolin	286.25	36.16	0.25	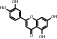	F
Jingjie	J2	Quercetin	302.25	46.43	0.28	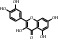	F
Jingjie	J3	beta-Sitosterol	414.79	36.91	0.75	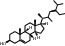	S
Jingjie	J4	Sitosterol	414.79	36.91	0.75	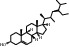	S
Jingjie	J5	Stigmasterol	412.77	43.83	0.76	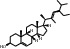	S
Jingjie	J6	Supraene	410.8	33.55	0.42	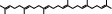	T
Jingjie	J7	Diosmetin	300.28	31.14	0.27	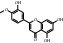	F
Jingjie	J8	Campest-5-en-3-beta-ol	400.76	37.58	0.71	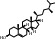	S
Jingjie	J9	J9	302.3	47.74	0.27	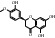	F
Jingjie	J10	Schizonepetoside B	330.42	31.02	0.28	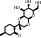	T
Jingjie	J11	Schkuhrin I	420.5	54.45	0.52	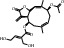	O
Niubangzi	N1	Neoarctin A	742.88	39.99	0.27	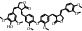	P
Niubangzi	N2	Arctiin	534.61	34.45	0.84	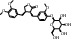	P
Niubangzi	N3	beta-Sitosterol	414.79	36.91	0.75	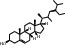	S
Niubangzi	N4	Kaempferol	286.25	41.88	0.24	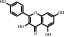	F
Niubangzi	N5	Supraene	410.8	33.55	0.42	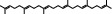	T
Niubangzi	N6	beta-Carotene	536.96	37.18	0.58	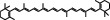	T
Niubangzi	N7	N7	386.48	52.3	0.48	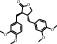	P
Niubangzi	N8	Cynarin(e)	516.49	31.76	0.68	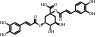	P
Baizhu	B1	B1	276.41	35.95	0.21	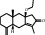	T
Baizhu	B2	B2	428.82	36.23	0.78	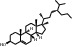	S
Baizhu	B3	*α*-Amyrin	426.8	39.51	0.76	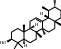	T
Baizhu	B4	3*β*-Acetoxyatractylone	274.39	54.07	0.22	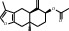	T
Baizhu	B5	B5	355.44	60.31	0.31	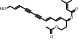	O
Baizhu	B6	B6	312.39	62.4	0.22	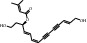	O
Baizhu	B7	B7	356.45	63.37	0.3	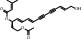	O

K22: (2R)-7-hydroxy-2-(4-hydroxyphenyl)chroman-4-one; J9: 5,7-dihydroxy-2-(3-hydroxy-4-methoxyphenyl)chroman-4-one; N7: (3R,4R)-3,4-bis[(3,4-dimethoxyphenyl)methyl]oxolan-2-one; B1: 8*β*-ethoxy atractylenolide III; B2: (24S)-24-propylcholesta-5-ene-3beta-ol; B5: 14-acetyl-12-senecioyl-2E,8E,10E-atractylentriol; B6: 12-senecioyl-2E,8E,10E-atractylentriol; B7: 14-acetyl-12-senecioyl-2E,8Z,10E-atractylentriol.

## Data Availability

The data used to support the findings of this study are included within the article.
